# Metformin Use on Incidence and Oncologic Outcomes of Bladder Cancer Patients With T2DM: An Updated Meta-Analysis

**DOI:** 10.3389/fphar.2022.865988

**Published:** 2022-04-07

**Authors:** Chen-Qian Liu, Jian-Xuan Sun, Jin-Zhou Xu, Xiao-Yuan Qian, Sen-Yuan Hong, Meng-Yao Xu, Ye An, Qi-Dong Xia, Jia Hu, Shao-Gang Wang

**Affiliations:** Department and Institute of Urology, Tongji Hospital, Tongji Medical College, Huazhong University of Science and Technology, Wuhan, China

**Keywords:** bladder cancer, metformin, meta-analyses, incidence, treatment outcome

## Abstract

**Background:** The incidence rate and mortality of bladder cancer are increasing year by year. Interestingly, the commonly used metabolic regulatory drug metformin has been reported to have anti-tumor effect in recent years. Nevertheless, it keeps unclear whether the usage of metformin is beneficial or unbeneficial in treating bladder cancer. Thus, a meta-analysis was conducted to explore the long-term effect of metformin on the incidence of bladder cancer and OS, PFS, DSS and RFS in bladder cancer patients with T2DM.

**Method:** We aim to collect evidence of the association between the usage of metformin and the incidence and treatment outcome of bladder cancer. We searched PubMed, Embase, Ovid Medline and Cochrane Library up to February 2021 to get effective literature reporting the effects of metformin in bladder cancer. The main outcomes were the protective effects of metformin on the incidence, overall survival (OS), recurrence-free survival (RFS), progression-free survival (PFS), and disease-specific survival (DSS) of bladder cancer. And OR (odds ratio) and HR (hazard ratio) with their 95%CI were pooled. Two independent researchers assessed the quality of included studies using the Newcastle-Ottawa Scale (NOS).

**Results:** We involved 12 studies meeting the inclusion criteria, including a total of 1,552,773 patients. The meta-analysis showed that use of metformin could decrease the incidence (OR = 0.45, 95%CI = 0.37–0.56; *p* < 0.01) and prolong recurrence-free-survival (HR = 0.56, 95%CI = 0.41–0.76; *p* = 0.91) of bladder cancer. However, there were no significant protective effects in the overall survival (HR = 0.93, 95%CI = 0.67–1.28, *p* = 0.05), disease-specific-survival (HR = 0.73, 95%CI = 0.47–1.16; *p* = 0.01), and progression-free-survival (HR = 0.78, 95%CI = 0.53–1.15, *p* = 0.34).

**Conclusion:** The results revealed that the usage of metformin could reduce the incidence of bladder cancer and prolong the prognosis of bladder cancer in T2DM patients, respectively. More prospective studies are needed to prove the protective role of metformin on bladder cancer.

## 1 Introduction

Metformin is a first-line oral metabolic regulatory medication for the treatment of type two diabetes mellitus (T2DM). It can reduce the production of glucose in the liver, increase the sensitivity of the body to insulin, and promote the utilization of glucose in peripheral tissues (Franciosi et al., 2013). Metformin is also used as a treatment for polycystic ovary syndrome related insulin resistance in non-diabetic patients (Diamanti-Kandarakis et al., 2010). Besides, the potential role of metformin in other aspects is also being explored.

In recent years, metformin has been reported to show an anti-cancer effects, especially on the prevention of various cancers, such as breast cancer, prostate cancer, colon cancer, pancreatic cancer, as well as bladder cancer (Yin et al., 2013). The anti-cancer mechanism of metformin can be divided into two types: one is related to insulin (direct anticancer pathway), and the other is not related to insulin (indirect anticancer pathway) (Ahn et al., 2020). The direct anticancer pathway is mainly related to the activation of AMPK and the inhibition of mTOR activity (Dowling et al., 2007). The indirect pathway also involves AMPK activation. By interfering with the gene transcription related to glucose production in hepatocytes, we can reduce glucose production, increase muscle glycogen decomposition, and then reduce insulin level and serum glucose (Shaw et al., 2005; Hardie et al., 2012).

Bladder cancer is the fourth most common cancers in men and is also common in women. It brings about 500,000 new cases and 200,000 deaths each year (Richters et al., 2020). The United States alone has more than 80,000 new cases and 17,000 deaths in a year (Siegel et al., 2019). The risk factors for bladder cancer are also apparent, including aging, men, smoking, and chemical exposure (Lenis et al., 2020). Also, as we know, bladder cancer can be divided into muscle-invasive bladder cancer (MIBC) and non-muscle-invasive bladder cancer (NMIBC). NMIBC patients usually have high recurrence rates and rapidly progress, while MIBC patients have a poor prognosis (Kirkali et al., 2005). Although there are many ways to treat bladder cancer at present, such as intravesical therapy, cystectomy, chemotherapy, radiotherapy, immunotherapy, and neoadjuvant therapy, the 5-year survival rate of bladder cancer is still difficult to improve (van den Bosch and Alfred Witjes, 2011; Chang et al., 2016; Ritch et al., 2020). Therefore, reducing the incidence and recurrence rate and improving survival rate have always been the focus of researchers.

Due to the long research and development cycle of new drugs, it has become a hot direction to break through the treatment of tumors through mature and safe listed drugs. Therefore, researchers have been working on the role of metformin in bladder cancer, but the protective effect of metformin reported in different cohort studies is different from that of bladder cancer. Therefore, it is necessary to perform a meta-analysis to explore the role of metformin in the pathogenesis and treatment of bladder cancer.

A meta-analysis of metformin and bladder cancer by Hu et al. (2018) has been reported in 2018, which suggested a surprising relationship between metformin and bladder cancer. While they chose OR (odds ratio) instead of HR (hazard ratio) as the effect size of prognosis, also they seemed to have extracted the wrong HR value from the included studies. Moreover, several new clinical studies have been published in the following years. Therefore, this meta-analysis is a correction and update of the previous one, and focus more on the effect of long-term use of metformin on OS, RFS, PFS, DSS of bladder cancer.

## 2 Methods

### 2.1 Search Strategy

The database search was conducted in February 2021. Two authors (Liu. and Xia) searched four databases including PubMed, Embase, Ovid Medline and Cochrane Library. Notably, Grey literature was searched in the American Society of Clinical Oncology (ASCO) conference abstract, the European Association of Urology (EAU) conference abstract, the American Urology Association (AUA) conference abstract, and pre-print databases, The detailed search strategies are described in Supplementary File S1. All abstracts and review articles about the subject were reviewed, and references in related meta-analyses were also reviewed. Besides, after completing the writing and revision of the manuscript, we conducted a repeated literature search to confirm there was no more updated literature need to be included.

### 2.2 Inclusion Criteria

All the studies included must meet the following PICOS (patients, interventions, comparators, outcomes, and study design) criteria:

• Patients: For oncologic outcomes of bladder cancer: diagnosed with bladder cancer (all tumor stage) and T2DM. For incidence of bladder cancer: diagnosed with T2DM, and some of them subsequently diagnosed as bladder cancer
• Interventions: metformin taken history in T2DM patients.
• Comparators: T2DM patients who did not take metformin (including patients who take other hypoglycemic drugs).
• Outcomes: incidence of bladder cancer or overall survival (OS); disease-specific survival (DSS); recurrence-free survival (RFS); progression-free survival (PFS).
• Study design: cohort studies with a controlled group or randomized controlled clinical trials.

### 2.3 Exclusion Criteria

Patients who used two or more hypoglycemic drugs containing metformin.

### 2.4 Data Extraction and Quality Assessment

Two authors (Liu and Xia) extracted data and information from final studies, such as the first author, year of publication, country of study, study type, tumor stage, follow-up period, age, and survival analysis. The quality of studies was evaluated by two authors independently using Newcastle-Ottawa Scale (NOS) (Supplementary File S2). Score of 7–9 was defined as high-quality, and <7 was defined as low-quality.

### 2.5 Statistical Analysis

We performed the meta-analysis with “meta” package in R v4.0.0 (Balduzzi et al., 2019). We pooled effect size as hazard ratio (HR) with 95% CIs (Tierney et al., 2007). When the HRs and their 95% CIs were available in the original articles, we extracted them directly. Otherwise, we estimated them based on the Kaplan-Meier survival curves or the related data according to the method described by Tierney et al. (2007). We used the standard Cochrane’s Q test and **I** ^2^ statistics to identify heterogeneity among the included studies. The value of **I** ^2^ statistics >50% and *p* < 0.1 indicated significant heterogeneity. When heterogeneity was significant, we then conducted the subgroup and sensitivity analysis. Besides, due to the small number of studies, we could not evaluate the publication bias by Egger test or Begg funnel plot (Begg and Mazumdar, 1994; Egger et al., 1997), but we could apply sensitivity analysis to estimate the stability of our meta-analysis.

## 3 Results

### 3.1 Search Results and Study Characteristics

**FIGURE 1:**
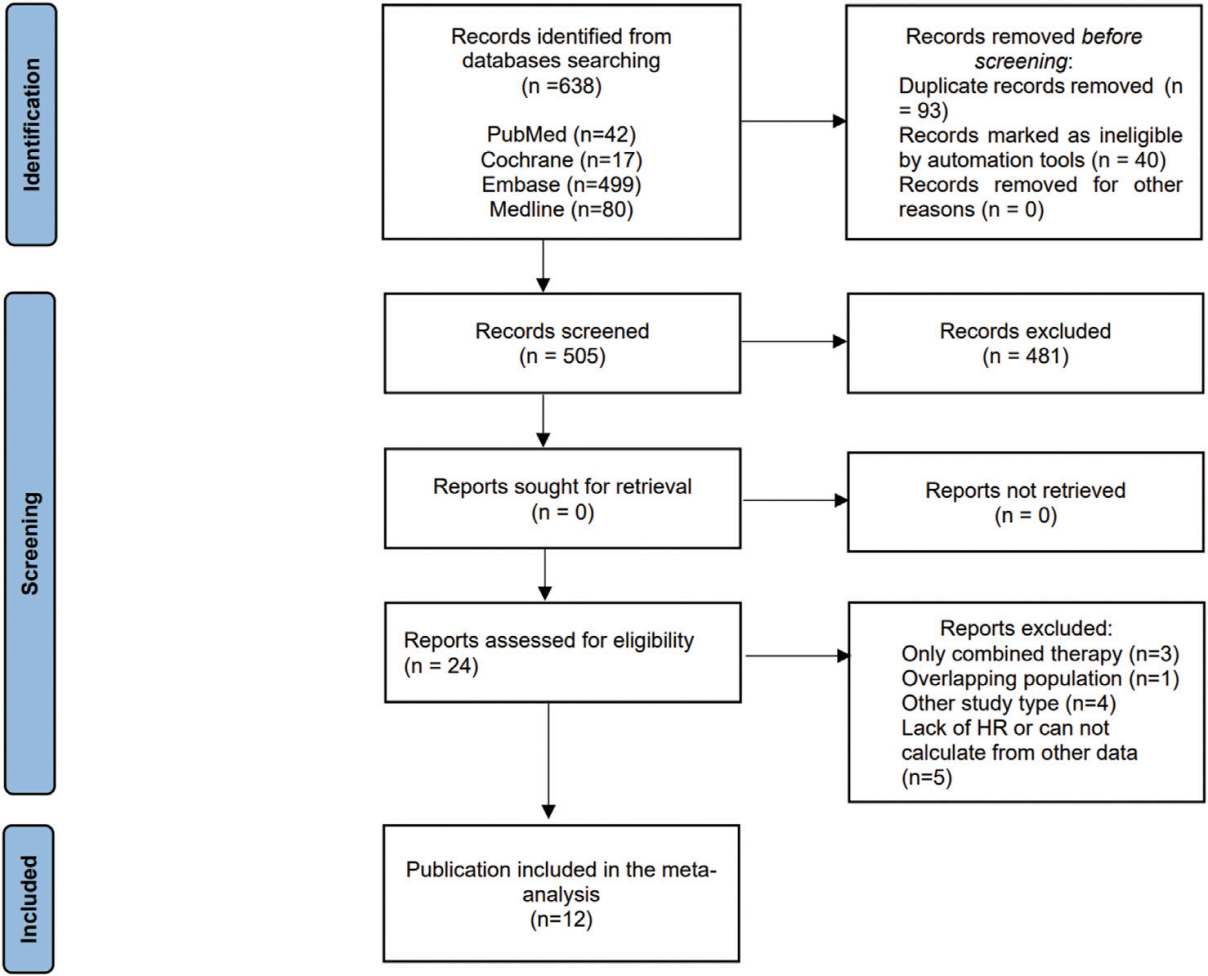
Flow chart of studies selection. HR = hazard ratio.

In total, 638 publications were identified initially through a literature search up to February 2021. After removing the duplicate studies and separately reviewing titles, abstracts, and the full texts, 12 studies with 1,555,074 individuals were included in our meta-analysis (Figure 1). Only the study by Wang et al. (2020) is a randomized controlled clinical trial, the other 11 studies are all cohort studies. The basic characteristics of all the included studies are shown in Table 1. Studies were published between 2013–2020, and there are 6, 5, 5, 4 studies related to the incidence of bladder cancer, metformin, and OS, metformin and RFS, metformin and DSS, respectively. As for the quality of included studies, NOS scores range from 7, 8. The detailed information is listed in the Supplementary Table S1.

**TABLE 1:** Basic characteristics of studies included.

Study (Year)	Country	Study type	Multicenter	NOS scores	Tumor stage	Treatment comparison	Follow-up (months)	Male patients (%)	Age	Survival analysis	Measure of outcomes
Rieken et al. (2013)	United States	Cohort study	Y	7	NMIBC	Metformin vs non-metformin	64	76.50%	65.0 (mean)	OS, RFS, PFS	HR
Mamtani et al. (2014)	United Kingdom	Cohort study	Y	8	NR	Metformin vs sulfonylurea (SU)	24	55.67%	62.0 in metformin, 69.0 in SU (median)	Incidence of bladder cancer	/
Tseng (2014)	Taiwan, China	Cohort study	Y	8	NR	Metformin vs non-metformin	NR	49.00%	NR	Incidence of bladder cancer	/
Rieken et al. (2014)	United States	Cohort study	Y	7	NMIBC + MIBC	Metformin vs non-metformin	34	78.40%	66.0 (median)	OS, RFS, DSS	HR
Tsilidis et al. (2014)	United Kingdom	Cohort study	Y	8	NR	Metformin vs sulfonylurea (SU)	61	56.59%	NR	Incidence of bladder cancer	/
Chen et al. (2015)	Taiwan, China	Cohort study	Y	8	NMIBC	Metformin vs sulfonylurea (SU)	30	53.80%	60.6 in metformin, 62.4 in SU (median)	Incidence of bladder cancer	/
Nayan et al. (2015)	Canada	Cohort study	N	7	NMIBC + MIBC	Metformin vs non-metformin	50	81.00%	71.0 (mean)	OS, RFS, DSS	HR
Richard (2018)	Canada	Cohort study	N	8	NMIBC	Metformin vs non-metformin	62.4	79.00%	78.0 (median)	OS, DSS	HR
Goossens et al. (2015)	United Kingdom	Cohort study	Y	8	NR	Metformin vs sulfonylurea (SU)	NR	53.13%	60.0 in metformin, 67.0 in SU (median)	Incidence of bladder cancer	/
Ahn et al. (2016)	Korea	Cohort study	Y	7	NMIBC	Metformin vs non-metformin	46	84.96%	64.6 (median)	RFS, PFS	OR
Wang et al. (2020)	Singapore	randomized controlled clinical trial	N	7	NMIBC	Metformin vs non-metformin	102	82.80%	64.7 (median)	OS, RFS, DSS	HR
Sung et al. (2020)	Hongkong, China	Cohort study	Y	8	NR	Metformin vs non-metformin	53	53.08%	NR	Incidence of bladder cancer	/

NMIBC, non-muscle-invasive bladder cancer; MIBC, muscle-invasive bladder cancer; OS, overall survival; DSS, disease-specific-survival; PFS, progression-free-survival; RFS, recurrence-free-survival; HR, hazard ratio; OR, odds ratio.

### 3.2 Metformin Intake and Bladder Cancer Incidence

**FIGURE 2:**
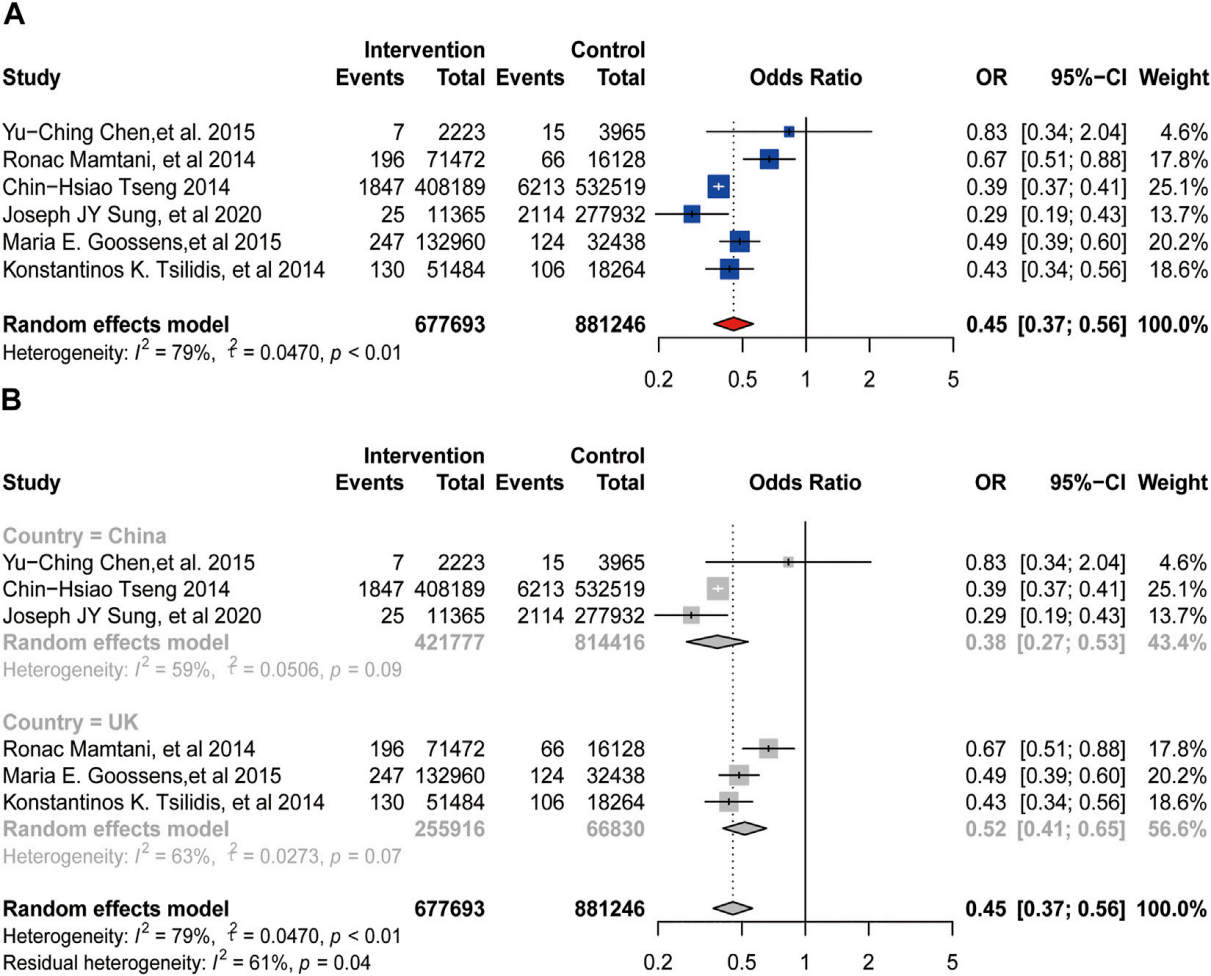
Forest plot of odds ratio for incidence **(A)** Association between use of metformin and incidence of bladder cancer. **(B)** Subgroup analysis based on country.

A total of six studies reported the relationship between metformin and the incidence of bladder cancer, including 1,552,773 patients. There was significant heterogeneity among these studies ( $I^{2}$ = 79%, *p* < 0.01, Figure 2A), so we used the random-effects model in our analysis. Heterogeneity may be caused by different countries, as well as the small number of the included studies. Therefore, we conducted a subgroup analysis based on country. Overall, the intake of metformin was associated with the reduced incidence of bladder cancer (OR = 0.45, 95%CI = 0.37–0.56; *p* < 0.01). The subgroup analysis by different countries showed that metformin use was related to the reduced incidence of bladder cancer among British (OR = 0.52, 95%CI = 0.41–0.65; *p* = 0.07) and Chinese (OR = 0.38, 95%CI = 0.27–0.53; *p* < 0.09) (Figure 2B).

### 3.2 Metformin Intake and the Oncologic Outcomes of Bladder Cancer

A total of 5, 4, 2, four studies provided the HR and their 95%CIs about the association between metformin use and the OS, DSS, PFS, and RFS of bladder cancer, respectively, which involved 2174, 2049, 252, 537 patients, respectively. If significant heterogeneity was found, we then used random-effects model for analysis and conducted subgroup analysis based on the sources of heterogeneity we speculated, otherwise we used fixed-effects model for analysis.

#### 3.2.1 Metformin and Overall Survival, Disease-Specific Survival

**FIGURE 3:**
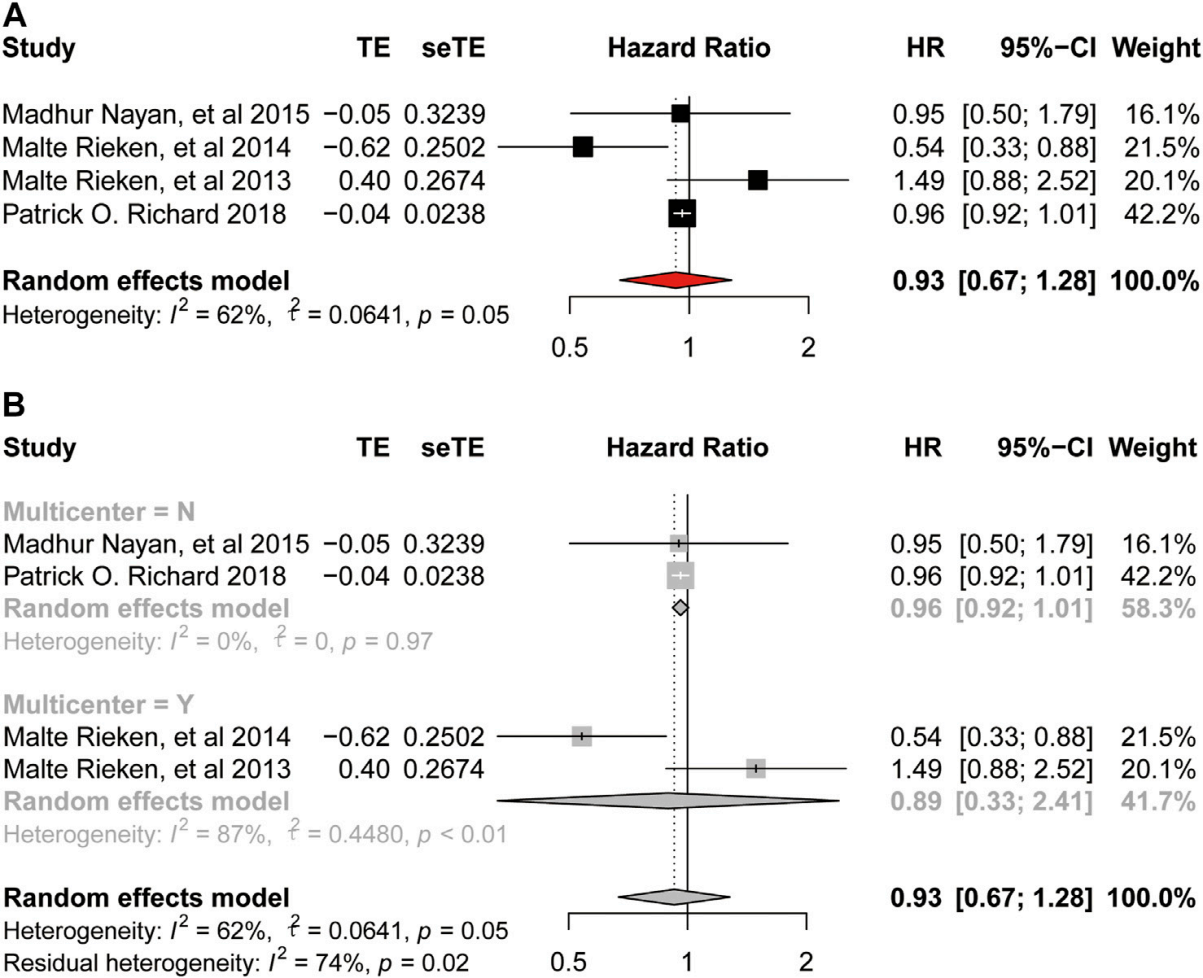
Forest plot of hazard ratio for overall survival **(A)** Association between use of metformin and OS of bladder cancer. **(B)** Subgroup analysis based on whether it is multicenter.

**FIGURE 4:**
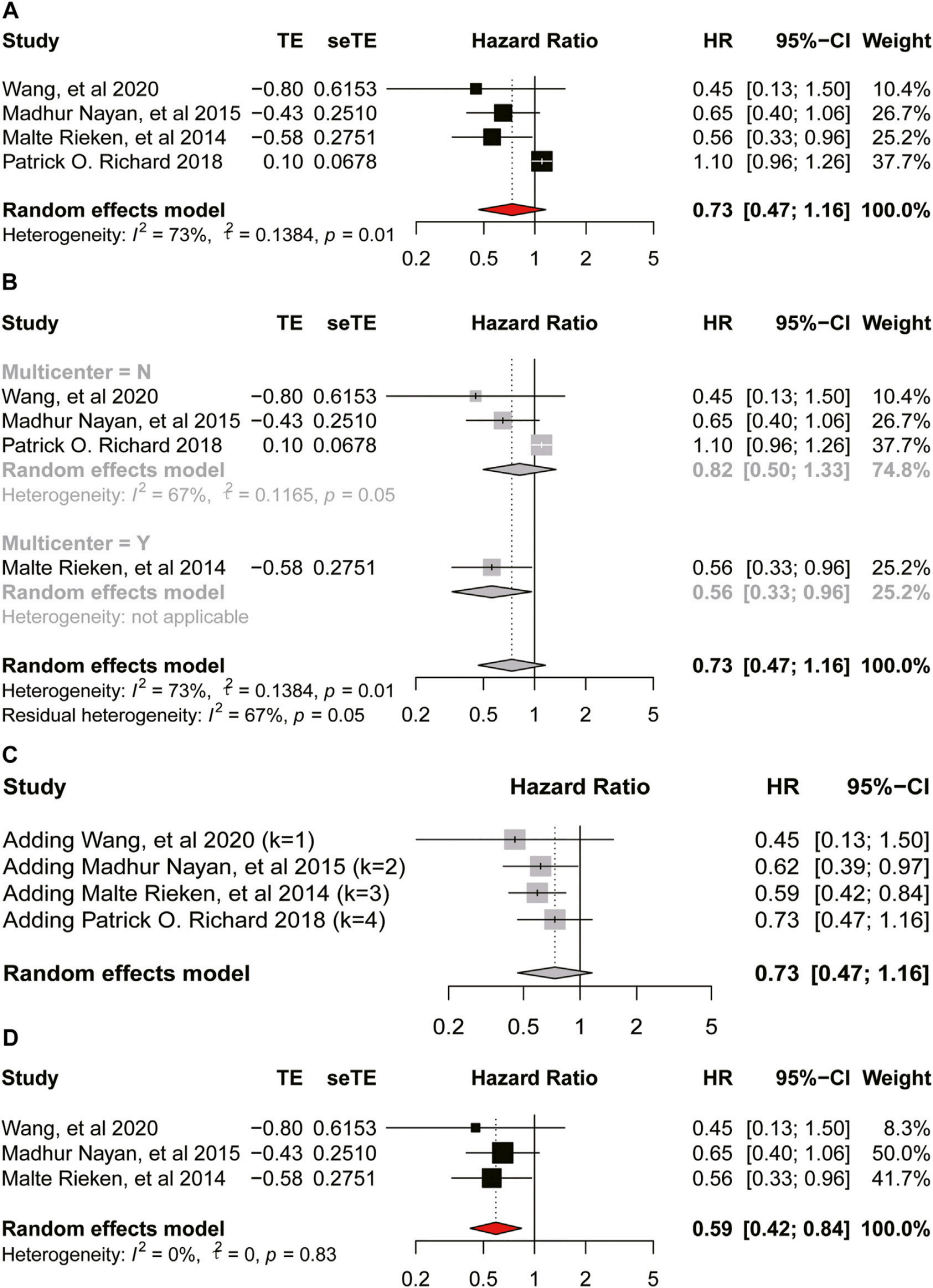
Forest plot of hazard ratio for disease-specific-survival **(A)** Association between use of metformin and DSS of bladder cancer. **(B)** Subgroup analysis based on whether it is multicenter. **(C)** Accumulation meta-analysis. **(D)** Association between use of metformin and DSS of bladder cancer after removing a study.

There was significant heterogeneity among these studies provided OS ( $I^{2}$ = 62%, *p* = 0.05, Figure 3A) and DSS ( $I^{2}$ = 73%, *p* = 0.01, Figure 4A). Thus, we did a subgroup analysis according to whether a multicenter study was performed. In general, there was no significant correlation between bladder cancer and OS (HR = 0.93, 95%CI = 0.67–1.28, *p* = 0.05, Figure 3A), and neither in the multicenter group (HR = 0.89, 95%CI = 0.33–2.41, *p* < 0.01, Figure 3B) nor the non-multicenter group (HR = 0.96, 95%CI = 0.92–1.01; *p* = 0.97, Figure 3B) in the subgroup analysis. The result of sensitivity analysis showed no obvious change to the HR value (Figure 3C). Therefore, our meta-analysis is stable. Meanwhile, metformin intake was proved to have no significant association with DSS of bladder cancer (HR = 0.73, 95%CI = 0.47–1.16; *p* = 0.01, Figure 4A) and neither in the non-multicenter group (HR = 0.82, 95%CI = 0.50–1.33, *p* = 0.05, Figure 4B). Moreover, although the result of the multicenter group was significant, this result was regarded as inaccurate because this group contained only one study. Later we did an accumulation meta-analysis (Figure 4C) and sensitivity analysis to analyze the outlier result among the four studies and found that the study Richard et al. (2018) was the one. So, we omitted this study and conducted the meta-analysis again using the left three studies. Here in Figure 4D, metformin intake was a protective factor for the DSS of bladder cancer ( $I^{2}$ = 0%; HR = 0.59, 95%CI = 0.42–0.84; *p* = 0.83).

#### 3.2.2 Metformin and Progression-Free Survival, Recurrence-Free Survival

**FIGURE 5:**
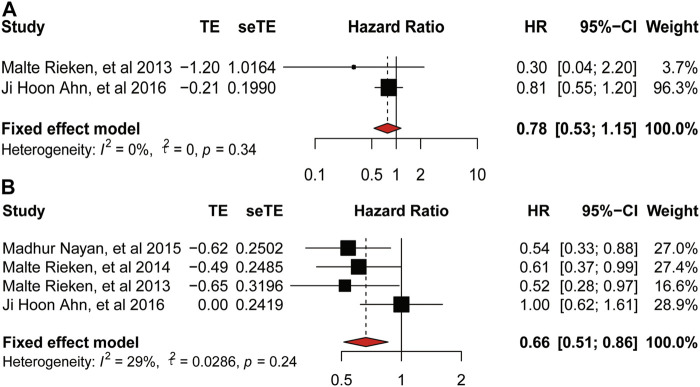
Forest plot of hazard ratio for progression-free-survival and recurrence-free-survival. **(A)** Association between use of metformin and PFS of bladder cancer. **(B)** Association between use of metformin and RFS of bladder cancer.

There was no heterogeneity among studies provided PFS ( $I^{2}$ = 0%, *p* = 0.34, Figure 5A) and RFS ( $I^{2}$ = 0%, *p* = 0.91, Figure 5B). But we did not conduct the subgroup analysis due to the small number of involved studies. And the use of metformin was not associated with the PFS of bladder cancer (HR = 0.78, 95%CI = 0.53–1.15, *p* = 0.34, Figure 5A), while it was a significant protective factor (HR = 0.56, 95%CI = 0.41–0.76; *p* = 0.91, Figure 5B) for RFS.

### 3.3 Sensitivity Analysis of Involved Studies

We used leave-one-out validation in the sensitivity analysis to find the sources of heterogeneity. Except for the Richard et al. (2018) we mentioned in the DSS part, all the other studies showed good stability. Therefore, we specifically excluded Richard et al. and conducted a meta-analysis again. So, our meta-analysis was convincing and stable, respectively (Supplementary Figure S1).

## 4 Discussion

In this meta-analysis, we included 12 retrospective cohort studies on the association between the usage of metformin and the incidence or oncologic outcomes of bladder cancer. And we chose to use OR (odds ratio) and HR (hazard ratio) as our effect sizes. OR is often used to analyze the correlation between exposure risk factors and disease (or medication). It is mainly an index reflecting the intensity of association between exposure (metformin use) and disease (bladder cancer). And HR is used to indicate the difference in risk between the experimental group (metformin use) and the control group (non-metformin use). As for the incidence of bladder cancer, three studies (Tseng, 2014; Chen et al., 2015; Sung et al., 2020) reported that the use of metformin might have a protective effect on the incidence of it, while the other three studies (Mamtani et al., 2014; Tsilidis et al., 2014; Goossens et al., 2015) demonstrated that there was no association between the intake of metformin and it. To our surprise, when we put the six studies altogether for analysis, metformin turned to be a significant protective factor for the incidence of bladder cancer (OR = 0.45, 95%CI = 0.37–0.56; *p* < 0.01), which is different from the result shown in the latest meta-analysis conducted by Hu et al. (2018). We conjectured the reason is that we added a newly published study for analysis (Sung et al., 2020), and we used OR value instead of HR value as the effect size for evaluation. HR value is mainly used for survival analysis and is not suitable for analyzing the relationship between exposure and morbidity. However, during the analysis, there was obvious heterogeneity among the studies, and the subgroup analysis based on country did not reduce the heterogeneity. We speculated the source of heterogeneity might occur from different tumor stages of included patients, while some of the studies did not offer this information. Thus, we conducted sensitivity analysis to detect heterogeneity, and the results did not change, indicating that our results are stable and reliable.

As for the oncologic outcomes of bladder cancer, the result of meta-analysis on RFS also indicated low heterogeneity and the use of metformin could improve the RFS of bladder cancer. Moreover, we could infer that our meta-analysis was stable according to the result of sensitivity analysis. Besides, we found no relationship between the intake of metformin and DSS of bladder cancer (HR = 0.73, 95%CI = 0.47–1.16; *p* = 0.01). While, when we removed the study by Richard et al. according to the result of sensitivity analysis, result of the second analysis showed a reduced heterogeneity and use of metformin on the DSS ( $I^{2}$ = 0%; HR = 0.59, 95%CI = 0.42–0.84, *p* = 0.83). We found that the proportion of male patients and the distribution of patients’ residence (urban or rural) were significantly different when comparing the baseline characteristics of the metformin use group and non-metformin use group in the study by Richard et al. (2018). The authors subdivided metformin use into the pre-bladder-cancer exposure and post-bladder-caner exposure, indicating the study was not suitable for inclusion in the analysis. In addition, although using metformin was not associated with OS and PFS, not all results from clinical studies were negative, so we need more high-quality evidence to determine the final role of metformin.

Bladder cancer is one of the most common urinary tract cancers, and its incidence rate and the mortality rate remain stubbornly high (Feng et al., 2019). We have already known the main causes of the disease. Besides the avoidable risk factors (smoking and exposure to chemical agents), we need other ways to prevent the occurrence of bladder cancer for people who can’t avoid risk exposure. Metformin, an early-used, well-researched metabolic regulatory medicine for first-line T2DM treatment, has been widely included in the research of various anticancer treatments in recent years (Janzer et al., 2014). The concept of “new use of old drugs” is also gradually known by researchers. Here in our meta-analysis, the intake of metformin could reduce the incidence of bladder cancer in T2DM patients, indicating that metformin can be a common drug for high-risk bladder cancer patients with T2DM to prevent bladder cancer. In addition, although there are now different ways to treat bladder cancer, the high recurrence rate is still a common problem for researchers. Reducing the recurrence rate can not only prolong the life of patients, but also reduce the fear of disease progression and improve their quality of life.

Fortunately, more and more researchers begin to pay attention to the relationship between metformin and bladder cancer, and continue to study the deep mechanism. For example, Deng et al. (2021) revealed the SREBP-1c/FASN axis using bladder cancer cell lines, also Wu et al. (2019) showed that metformin targets a YAP1-TEAD4 complex *via* AMPKα, which is the classic anti-tumor pathway for metformin, and to regulate CCNE1/2 in bladder cancer cells. As for clinical studies, our meta-analysis showed that metformin could prolong the RFS of patients with bladder cancer, which undoubtedly provided a new way of thinking for the treatment of bladder cancer. In the future, we also need more clinical studies to constantly confirm the role of metformin and continue to improve it.

There are many limitations. First, our meta-analysis only included the patients who diagnosed with T2DM, which is not possible to extend the anticancer effect of metformin to the whole bladder cancer population. Second, since most studies did not provide information on tumor stage and dose of metformin use, we did not analyze these two factors. The different tumor stage and dose of metformin use could be confounding factor. Third, some HR values were extracted according to the survival curve, which would cause some errors. Therefore, we extracted three times and averaged the values in order to reduce the influence. Forth, we only included English language studies, which would lead to publication bias, while due to the small number of included studies, we could not evaluate publication bias. Fifth, the most of the literatures we included were all cohort studies, and the level of evidence is lower than that of RCT (random controlled trials) studies, which would also bring some selection bias. Last, considering the difficulty of obtaining articles, we did not retrieve unpublished studies. So we are waiting for more clinical studies that including the non-T2DM patients and more RCT in high quality that we could get a more convincing result.

## 5 Conclusion

This meta-analysis demonstrated that the use of metformin could reduce the incidence and improve the RFS of bladder cancer patients with T2DM. Although no significant association between the OS, DSS, PFS and bladder cancer was found, some clinical studies still showed a positive attitude. Therefore, this is a significant need for more clinical studies to prove the role of metformin in bladder cancer.

## Data Availability

The original contributions presented in the study are included in the article/Supplementary Material, further inquiries can be directed to the corresponding authors.
