# Supplementary material for: Metformin Use on Incidence and Oncologic Outcomes of Bladder Cancer Patients With T2DM: An Updated Meta-Analysis
Source: Front Pharmacol. 2022 Apr 7;13:865988. doi: 10.3389/fphar.2022.865988 (PMC9021395; doi:10.3389/fphar.2022.865988)
Supplement: Supplementary file 1 [file Table1.doc]

**Supplementary Table 1**. The Newcastle-Ottawa Scale (NOS) for assessing the quality of cohort studies.

| **Study (year)** | **Seletion** | | | | **Comparability** | | **Assessment of outcome** | | | **Total quality score** |
| --- | --- | --- | --- | --- | --- | --- | --- | --- | --- | --- |
|  | **Representative-ness of the exposed cohort** | **Ascertainment of exposure** | **Selection of Controls** | **Demonstration that outcome of interest was not present at start of study** | **study controls for the most important factors** | **study controls for any additional factor** | **Assessment of outcome** | **Was follow-up long enough for outcomes to occur** | **Adequacy of follow up of cohort** |  |
| Rieken (2013) | * | * | * | / | * | * | * | * | / | 7 |
| Mamtani (2014) | * | * | * | / | * | * | * | * | * | 8 |
| Rieken (2014) | * | * | * | / | * | * | * | * | / | 7 |
| Tsilidi (2014) | * | * | * | / | * | * | * | * | * | 8 |
| Tseng (2014) | * | * | * | / | * | * | * | * | * | 8 |
| Goossens (2015) | * | * | * | / | * | * | * | * | * | 8 |
| Chen (2015) | * | * | * | / | * | * | * | * | * | 8 |
| Nayan (2015) | * | * | * | / | * | * | * | * | / | 7 |
| Richard (2015) | * | * | * | / | * | * | * | * | * | 8 |
| Ahn (2016) | * | * | * | / | * | * | * | * | / | 7 |

| **Study (year)** | **Seletion** | | | | **Comparability** | | **Assessment of outcome** | | | **Total quality score** |
| --- | --- | --- | --- | --- | --- | --- | --- | --- | --- | --- |
|  | **Representative-ness of the exposed cohort** | **Ascertainment of exposure** | **Selection of Controls** | **Demonstration that outcome of interest was not present at start of study** | **study controls for the most important factors** | **study controls for any additional factor** | **Assessment of outcome** | **Was follow-up long enough for outcomes to occur** | **Adequacy of follow up of cohort** |  |
| Wang (2020) | * | * | * | / | * | * | * | * | / | 7 |
| Sung (2020) | * | * | * | / | * | * | * | * | * | 8 |
