# Supplementary material for: Metformin Use on Incidence and Oncologic Outcomes of Bladder Cancer Patients With T2DM: An Updated Meta-Analysis
Source: Front Pharmacol. 2022 Apr 7;13:865988. doi: 10.3389/fphar.2022.865988 (PMC9021395; doi:10.3389/fphar.2022.865988)
Supplement: Supplementary file 2 [file DataSheet1.docx]

**Pubmed: 42**

#1 Urinary Bladder Neoplasms[MeSH Terms] 54935

#2 (((((((((((((((((Neoplasm, Urinary Bladder[Title/Abstract]) OR (Urinary Bladder Neoplasm[Title/Abstract])) OR (Neoplasms, Bladder[Title/Abstract])) OR (Bladder Neoplasms[Title/Abstract])) OR (Bladder Neoplasm[Title/Abstract])) OR (Neoplasm, Bladder[Title/Abstract])) OR (Bladder Tumors[Title/Abstract])) OR (Bladder Tumor[Title/Abstract])) OR (Tumor, Bladder[Title/Abstract])) OR (Tumors, Bladder[Title/Abstract])) OR (Urinary Bladder Cancer[Title/Abstract])) OR (Cancer, Urinary Bladder[Title/Abstract])) OR (Malignant Tumor of Urinary Bladder[Title/Abstract])) OR (Cancer of the Bladder[Title/Abstract])) OR (Bladder Cancer[Title/Abstract])) OR (Bladder Cancers[Title/Abstract])) OR (Cancer, Bladder[Title/Abstract])) OR (Cancer of Bladder[Title/Abstract])

#3 #1 OR #2 66214

#4 Metformin[MeSH Terms] 13733

#5 (((((Dimethylbiguanidine[Title/Abstract]) OR (Dimethylguanylguanidine[Title/Abstract])) OR (Glucophage[Title/Abstract])) OR (Metformin Hydrochloride[Title/Abstract])) OR (Hydrochloride, Metformin[Title/Abstract])) OR (Metformin HCl[Title/Abstract])

#6 #4 OR #5 13942

#7 #3 AND #6 42

**Cochrane 17**

#1 MeSH descriptor: [Metformin] explode all trees 4111

#2 (metformin):ti,ab,kw OR (Dimethylbiguanidine):ti,ab,kw OR (Dimethylguanylguanidine):ti,ab,kw OR (Glucophage):ti,ab,kw OR (Metformin Hydrochloride):ti,ab,kw OR (Hydrochloride, Metformin):ti,ab,kw OR (Metformin HCl):ti,ab,kw OR (HCl, Metformin):ti,ab,kw 10686

#3 #1 OR #2 10686

#4 MeSH descriptor: [Urinary Bladder Neoplasms] explode all trees 1471

#5 (Urinary Bladder Neoplasms):ti,ab,kw OR (Neoplasm, Urinary Bladder):ti,ab,kw OR (Urinary Bladder Neoplasm):ti,ab,kw OR (Neoplasms, Bladder):ti,ab,kw OR (Bladder Neoplasms):ti,ab,kw OR (Bladder Neoplasm):ti,ab,kw OR (Neoplasm, Bladder):ti,ab,kw OR (Bladder Tumors):ti,ab,kw OR (Bladder Tumor):ti,ab,kw OR (Tumor, Bladder):ti,ab,kw OR (Tumors, Bladder):ti,ab,kw OR (Urinary Bladder Cancer):ti,ab,kw OR (Cancer, Urinary Bladder):ti,ab,kw OR (Malignant Tumor of Urinary Bladder):ti,ab,kw OR (Cancer of the Bladder):ti,ab,kw OR (Bladder Cancer):ti,ab,kw OR (Bladder Cancers):ti,ab,kw OR (Cancer, Bladder):ti,ab,kw OR (Cancer of Bladder):ti,ab,kw 5105

#6 #4 OR #5

#7 #3 AND #6 17

**Embase 499**

#1 bladder cancer.mp. or exp bladder cancer/ 82695

#2 (urinary bladder cancer or urine bladder cancer or vesical cancer or bladder tumor or urinary tract cancer or bladder carcinogenesis or bladder metastasis or muscle invasive bladder cancer or non muscle invasive bladder cancer).ab,kw,ti. 18155

#3 #1 OR #2 85720

#4 metformin.mp. or exp metformin/ 68649

#5 (apophage or aron or benofomin or dabex or denkaform or deson or dextin or diabetase or diabetases or diabetformin or diabetmin or diabetminretard or diabetosan or diabex or diafat or diaformin or diaformina or diaforminalp or diametin or diamin or dianben or diformin or diforminretard or dimefor or dimethylbiguanide or dimethyldiguanide or dmgg or dybis or eraphage or euformretard or fluamine or flumamine or fornidd or fortamet or glafornil or glibudon or glifage or gliguanid or glucaminol or glucofage or glucofago or glucoform or glucoformin or glucohexal or glucoless or glucomet or glucomin or glucomine or gluconil or glucophage or glucophageforte or glucophageretard or glucophagesr or glucophagexr or glucophagexrextendedrelease or glucostop or glucotika or gludepatic or glufor or gluformin or glukophage or glumeformin or glumet or glumetza or glupa or glustress or glyciphage or glycomet or glycon or glycoran or glyformin or glymet or haurymellin or hipoglucin or islotin or juformin or maformin or meglucon or meguan or melbin or melformin or mellittin or merckformin or mescorit or metaformin or metfogamma or metfoliquidgeriasan or metforal or metformax or metforminhydrochloride or metformina or metformine or metforminehcl or methformin or metiguanide or metomin or metphormin or miformin or n'dimethylguanylguanide or n'dimethylguanylguanidine or n,ndimethylbiguanidine or n',n'dimethyldiguanide or n,ndimethylbiguanide or n,ndimethylbiguanideretard or n,ndimethylguanylguanidine or n,ndimethylbiguanidine or neoform or nndg or riomet or riomet or riometer or risidon or siamformet or siofor or thiabet or vimetrol or biguanide derivative or walaphage or hydroxymethylglutaryl coenzyme A reductase kinase activator or oral antidiabetic agent).ab,kw,ti. 1412

#6 #4 OR #5

#7 #3 AND #6 499

**Medline: 80**

#1 Urinary Bladder Neoplasms.mp. or exp Urinary Bladder Neoplasms/ 55206

#2 (Urinary Bladder Neoplasms or Neoplasm, Urinary Bladder or Urinary Bladder Neoplasm or Neoplasms, Bladder or Bladder Neoplasms or Bladder Neoplasm or Neoplasm, Bladder or Bladder Tumor or Bladder Tumors or Tumor, Bladder or Tumors, Bladder or Cancer, Urinary Bladder or Urinary Bladder Cancer or Malignant Tumor of Urinary Bladder or Cancer of the Bladder or Bladder Cancer or Bladder Cancers or Cancer, Bladder or Cancer of Bladder).ab,ti. 41151

#3 #1 or #2 66047

#4 metformin.mp. or exp Metformin/ 23116

#5 (metformin or Dimethylbiguanidine or Dimethylguanylguanidine or Glucophage or Metformin Hydrochloride or Hydrochloride, Metformin or Metformin HCl or HCl, Metformin).ab,ti. 21038

#6 #4 or #5 23130

#7 #3 and #6 80
